# Use of C-Reactive Protein in Global Leadership Initiative on Malnutrition (GLIM) Etiologic Criteria for Critically Ill Patients: A Retrospective Claims Database Study

**DOI:** 10.3390/nu17040705

**Published:** 2025-02-16

**Authors:** Shinya Suganuma, Naoki Kanda, Minoru Yoshida, Tomoka Miyagi, Kensuke Nakamura

**Affiliations:** 1 Critical Care Medicine, Yokohama City University Hospital, Yokohama 236-0004, Kanagawa, Japan; uz.831@icloud.com (S.S.); 10mtn.3@gmail.com (T.M.); 2 Emergency and Critical Care Medicine, Hitachi General Hospital, Hitachi 317-0077, Ibaraki, Japan; naok16knd@gmail.com; 3 Division of General Internal Medicine, Jichi Medical University, Shimotsuke 329-0431, Tochigi, Japan; 4 Department of Emergency and Critical Care Medicine, St. Marianna University School of Medicine, Kawasaki 216-8511, Kanagawa, Japan; minoru.yoshida@marianna-u.ac.jp

**Keywords:** critical care, Global Leadership Initiative on Malnutrition, C-reactive protein, nutritional assessment, inflammation

## Abstract

**Background/Objectives**: The Global Leadership Initiative on Malnutrition (GLIM) is suggested by major societies. The etiologic criteria for inflammation in critically ill patients remain unclear. Because an initial nutritional assessment is recommended within 48 h, it is also possible to use C-reactive protein (CRP) up to 3 days after admission. The purpose of the present study is to explore the utility of CRP in identifying malnutrition and to determine whether a nutritional assessment incorporating CRP criteria can effectively identify malnourished patients in the intensive care unit (ICU). **Methods**: This was a retrospective cohort study of ICU patients. The primary outcome was a composite of in-hospital mortality, Barthel index < 60 at discharge, and length of hospital stay of 14 days or more. The area under the curve (AUC) for the primary outcome was calculated using CRP between days 0 and 2. We divided the patients into four groups using inflammation criteria with the optimal cut-off and low body mass index (BMI) criteria of the GLIM: CRP+/−, and BMI+/−. **Results**: A total of 38,981 patients were included. The AUC of the highest CRP between days 0 and 2 was 0.65, which was higher than the CRP on day 0 and the highest CRP between days 0 and 1 (0.59 and 0.63). The AUC and optimal cut-offs varied depending on diagnoses, with a maximum of 0.75 in neurology. The optimal cut-off for the maximum CRP was 3.82 mg/dL. In the four groups of CRP+BMI+, CRP+BMI-, CRP-BMI+, and CRP-BMI-, the in-hospital mortality values were 22.7, 14.4, 10.8, and 4.8% (*p* < 0.001 between all the groups). **Conclusions**: In an initial nutritional assessment of critically ill patients, it would be appropriate to use the maximum CRP over 3 days from ICU admission.

## 1. Introduction

Hospital malnutrition is an increasingly recognized challenge in healthcare, exerting a substantial impact on both patient outcomes and healthcare systems worldwide. It is estimated to affect approximately 20% to 50% of hospitalized patients [1], with the highest prevalence observed among those individuals admitted to medical units, particularly elderly patients [2], surgical patients [3], and oncology patients [4], who are at an elevated risk due to their complex medical needs. Furthermore, another population with a notably high prevalence of malnutrition includes patients in critical care settings, such as intensive care units (ICUs), where the severity of illness and metabolic demands contribute to a heightened nutritional risk [5].

A nutritional assessment to identify malnutrition is clinically important, and the Global Leadership Initiative on Malnutrition (GLIM) is recommended for this purpose. Many issues associated with the GLIM for critically ill patients have yet to be resolved. Nevertheless, previous studies validated the GLIM for critically ill patients [6,7], and, thus, the GLIM may be more actively applied to critically ill patients in the future. The GLIM comprises phenotypic and etiologic criteria, both of which may be evaluated in critically ill patients.

An inflammatory assessment has not yet been established in the GLIM etiologic criteria [8,9], particularly for critically ill patients. Since critically ill patients have serious diseases as a background, it has been proposed that the etiologic criteria are positive for the disease burden/inflammation. In other words, all critically ill patients need to be regarded as positive for nutritional screening and undergo a nutritional assessment [10]. The Nutrition Risk in Critically Ill (NUTRIC) score [11], known as a nutritional assessment in the intensive care unit (ICU), uses interleukin-6 (IL-6) as an inflammation criterion. Cytokines like IL-6 are sometimes measured for research purposes, but their testing is costly and available only in a limited number of facilities equipped for rapid testing. Consequently, the use of IL-6 and similar inflammatory markers in routine clinical practice is limited, making their application in daily nutritional assessments impractical. In contrast, C-reactive protein (CRP) is frequently measured at a lower cost in many countries. We believe that CRP warrants broader implementation in nutritional assessment, including integration into the GLIM framework. However, CRP has a long half-life and generally reaches its peak level 36 h after disease onset [12]. Therefore, several barriers need to be overcome before the application of CRP to a nutritional assessment.

CRP on the first day of admission has often been used as an indicator of inflammation [13]. However, the significance of CRP is determined by examining the trend over several days in actual clinical practice. To the best of our knowledge, changes in CRP levels over several days have not yet been examined as a predictor of outcomes, particularly in a nutritional assessment. A nutritional assessment in the ICU is recommended within 48 h [14,15]. Therefore, it may be possible to expand the use of CRP from the first day of admission to the first two or three days for a nutritional assessment based on its half-life and peak, which may lead to a breakthrough in nutritional assessments, including the GLIM.

In the present study, we used CRP from day 0 (on ICU admission) to day 2 of an ICU stay when a nutritional assessment was performed and examined the utility of CRP for identifying malnutrition and a poor prognosis. We also investigated whether a nutritional assessment combining the body mass index (BMI) criteria of the GLIM and the CRP criterion of the present study appropriately identified patients with malnutrition.

## 2. Materials and Methods

### 2.1. Data Source

The present study was a retrospective cohort design using the multicenter inpatient administrative claims database (Diagnosis Procedure Combination database [16]) with laboratory test values in Japan provided by Medical Data Vision Co., Ltd. (Chiyoda, Tokyo, Japan). Our database contains data on patients admitted to the ICU at Japanese acute care hospitals between March 2010 and September 2021. The present study was conducted in accordance with the Declaration of Helsinki and was approved by the Ethics Committee of Hitachi General Hospital (2020-131). The requirement for informed consent was waived due to its retrospective design and the use of anonymized data.

### 2.2. Study Population

We targeted patients ≥18 years who were emergently admitted to the ICU. Since a nutritional assessment within 48 h is recommended for critically ill patients, we enrolled patients who had been in the ICU for at least 3 consecutive days. In addition, to exclude patients who were discharged early, we examined patients who had been in hospital for at least five days, including the ICU. Exclusion criteria were as follows: (1) missing variables for the Barthel index (BI) at discharge, BMI, or age; (2) CRP levels not measured within 7 days. The day of admission was defined as day 0. In addition to patient backgrounds, such as age, sex, height, weight, and medical history, diagnoses for ICU admission and data on days 0–1, including continuous hemodialysis and filtration (CHDF), intermittent infusion hemodialysis, mechanical ventilation, extracorporeal membrane oxygenation (ECMO), and intra-aortic balloon pumping (IABP), were extracted. The Sequential Organ Failure Assessment (SOFA) score was calculated based on laboratory results and interventions on days 0–1. Since information on oxygenation (PaO2 and FiO2) was not included in our database, the respiratory component of SOFA was defined as follows: patients who did not receive oxygen therapy as 0, patients who received oxygen therapy as 1, patients who received non-invasive positive pressure ventilation (including a high-flow nasal cannula) as 2, patients who received mechanical ventilation as 3, and patients who received ECMO therapy as 4. We defined the cardiovascular component of the SOFA score for patients who did not receive catecholamines as 0.

We calculated the dose of catecholamines based on the average dose (μg/kg/minute) on day 1 using the total amount of their prescriptions on that day. The catecholamine index was calculated by the sum of dopamine, dobutamine, and noradrenaline multiplied by 100 and adrenaline multiplied by 100 (i.e., the catecholamine index [μg/kg/min] = dopamine [μg/kg/min] + dobutamine [μg/kg/min] + noradrenaline [μg/kg/min] × 100 + adrenaline [μg/kg/min] × 100). Diagnoses for ICU admission were categorized as sepsis, cardiovascular, pulmonary, metabolic, neurology, trauma, digestive, and others. The ICD-10 codes of each category were defined according to a previous study using the same database [17]: cardiovascular, I00–99; pulmonary, J00–99; metabolic, E00–99; neurology, I60–69 and G00–99; trauma, S00–99, T00–19, T33–88, V00–99, W00–99, X00–99, and Y00–09; and digestive, K20–93. The diagnostic codes of sepsis were based on a previous study [18], and patients who had the code of sepsis were categorized as the sepsis group.

### 2.3. Outcomes

The primary outcomes were in-hospital mortality, BI < 60 at discharge, and length of hospital stay of 14 days or more. In the analysis of the receiver operating characteristic (ROC) curve to select the optimal cut-off level for CRP, we used a composite of these outcomes. Secondary outcomes were 14-day mortality, 28-day mortality, LOS as a continuous variable, and BI as a continuous variable.

### 2.4. Statistical Analysis

Since this was a retrospective study, CRP levels were not measured every day from days 0 to 2. Missing CRP levels from days 0 to 2 were linearly interpolated using data obtained between days 0 and 6 and analyzed in the “all-case analysis”. Patients without missing CRP levels from days 0 to 2 were analyzed in the “complete case analysis”. This linear interpolation method was adopted in a previous study [19]. We drew a ROC curve for the composite outcome using the following three CRP levels: (1) the level on day 0, (2) the highest level between days 0 and 1, and (3) the highest level between days 0 and 2. We calculated the area under the ROC curve (AUC), and the optimal cut-off level for CRP was selected using the Youden index. We stratified patients by their diagnosis for ICU admission and calculated AUC and the optimal cut-off in each subgroup. The Youden index was employed to determine optimal CRP cut-off values for each subgroup analysis. This index, defined as the maximum value of (sensitivity + specificity − 1) on the ROC curve, identifies the threshold that maximizes the discriminative ability of CRP. We then classified patients into four groups based on the optimal cut-off level for CRP calculated in the present study and the BMI cut-off in the GLIM. The BMI criteria used were the low BMI criteria in the GLIM (<18.5 for <70 years and <20 for ≥70 years) for Asians. If the BMI criteria were met, it was defined as BMI+, and, if the CRP criterion was met, it was defined as CRP+ (i.e., patients were classified into four groups: CRP+BMI+, CRP+BMI-, CRP-BMI+, and CRP-BMI-). We compared LOS, in-hospital mortality, 14-day mortality, 28-day mortality, and BI for each group. Differences between groups were compared using the Kruskal–Wallis test, and we examined the significance of differences between two groups using the Conover test. All statistical analyses were performed using Phyton 3.12.1.

## 3. Results

**Figure 1:**
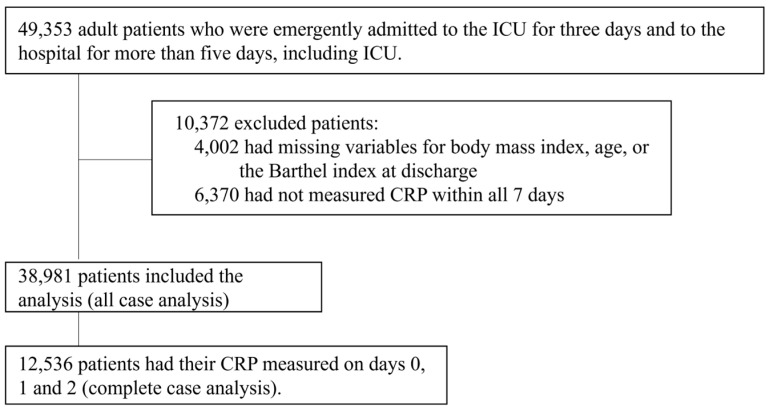
Patient flowchart. ICU, intensive care unit; CRP, C-reactive protein.

A total of 38,981 patients were included in the analysis (all-case analysis) after excluding 10,372 patients who met the exclusion criteria from the initial cohort of 49,353 patients who were emergently admitted to the ICU for 3 consecutive days and hospitalized for ≥5 days (Figure 1). Of the 38,981 patients, 12,536 (32%) for whom their CRP levels were measured every day between days 0 and 2 were included in the complete case analysis.

**Figure 2:**
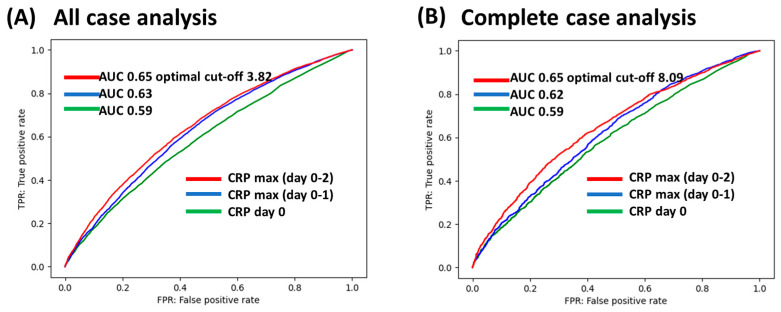
Receiver operating characteristic (ROC) curves of the maximum C-reactive protein value for the composite of in-hospital mortality, Barthel index < 60 at discharge, and length of hospital stay of 14 days or more. The results for (**A**) the all-case analysis and (**B**) complete case analysis are shown. The optimal cut-off level for CRP was calculated using the Youden index from the ROC curve for CRP max (days 0–2), which had the highest AUC. AUC, area under the curve; CRP, C-reactive protein.

A higher AUC was observed for the highest CRP level between days 0 and 2 compared to the CRP level on day 0 and the highest CRP level between days 0 and 1, both in the all-case analysis and the complete case analysis (Figure 2). The ROC curves for the composite outcome and AUCs were calculated using the CRP level on day 0, the highest CRP level between days 0 and 1, and the highest CRP level between days 0 and 2. In the all-case analysis, the AUCs were 0.59, 0.63, and 0.65, respectively. In the complete case analysis, the AUCs were 0.59, 0.62, and 0.65, respectively. The optimal cut-off was 3.82 mg/dL in the all-case analysis and 8.09 mg/dL in the complete case analysis.

The highest CRP level between days 0 and 2 showed a trend of higher AUCs for the primary outcome compared to the CRP level on day 0 and the highest CRP level between days 0 and 1 for each subgroup stratified by their diagnosis at ICU admission, both in the all-case analysis and the complete case analysis (Table 1). In the all-case analysis, the AUCs of all the subgroups were the highest when using the highest level between days 0 and 2. On the other hand, in the complete case analysis, the AUC was the highest using the level on day 0 for patients categorized as pulmonary and using the highest level between days 0 and 1 for those categorized as cardiovascular. In addition, the optimal cut-off differed depending on the diagnosis at ICU admission. In the all-case analysis, the optimal cut-off level for CRP was higher in the following groups: 15.65 mg/dL for sepsis; 10.29 mg/dL for digestive; and 5.52 mg/dL for pulmonary. On the other hand, the optimal cut-off level for neurology was lower than those for the other disease groups (0.76 mg/dL). In the all-case analysis, the optimal cut-off for the entire population, 3.82 mg/dL, had a sensitivity of 92% and specificity of 14% for those patients with sepsis, whereas it had a sensitivity of 31% and specificity of 94% for those patients with neurological disease (Supplementary Table S1). The complete case analysis showed similar results to those in the all-case analysis. The optimal cut-off levels were 20.6 mg/dL for sepsis, 15.46 mg/dL for digestive, 8.85 mg/dL for pulmonary, and 2.55 mg/dL for neurology.

When the patients were divided into four groups (CRP+BMI+, CRP+BMI-, CRP-BMI+, and CRP-BMI-) based on the BMI criteria of the GLIM and the CRP criterion (>3.82 mg/dL) in the all-case analysis, in the groups that met the CRP criterion (>3.82 mg/dL), there were higher percentages of patients receiving CHDF, mechanical ventilation, ECMO, and IABP, and their SOFA score and catecholamine index values were also higher, indicating higher illness severity (Table 2). The percentage of patients categorized under neurology at admission was 27.5% in the all-case analysis. The patients meeting the BMI criteria were older than those who did not. Male patients were more prevalent in the groups that did not meet the BMI criteria. The percentage of patients with sepsis was higher in the groups that met the CRP criterion: 22.7% in the CRP+BMI+ group. The percentage of patients categorized under neurology was higher in the groups that did not meet the CRP criterion: 46.3% in the CRP-BMI- group. The percentage of patients categorized under pulmonary was the highest in the CRP+BMI+ group (14.1% in CRP+BMI+ group).

The percentage of patients categorized under neurology at admission was 8.1% in the complete case analysis. In the complete case analysis, similar to the all-case analysis, the groups that met the BMI criteria were older, while the groups that did not meet the BMI criteria had a higher percentage of males. Similar to the all-case analysis, the percentage of patients receiving CHDF and mechanical ventilation was higher and the SOFA score and catecholamine index were also higher in the groups that met the CRP criterion. The percentage of patients with sepsis was higher in the groups that met the CRP criterion, while the percentage of patients categorized under neurology was higher in the groups that did not meet the CRP criterion (Supplementary Table S2).

The CRP+BMI+ group showed the worst prognosis, with the highest rates of in-hospital mortality (22.7%), BI < 60 (73.9%) and LOS ≥ 14 (86.2%) (Table 3). Significant differences were observed between any two groups for in-hospital mortality, BI < 60, and LOS ≥ 14 (all *p*-values < 0.01). The composite outcome did not significantly differ between the CRP+BMI- and CRP-BMI+ groups (*p* = 0.04). In-hospital mortality and the percentage of patients with LOS ≥ 14 were higher in the order of CRP+BMI+, CRP+BMI-, CRP-BMI+, and CRP-BMI- (in-hospital mortality: 22.7% vs. 14.4% vs. 10.8% vs. 4.8%; percentage of patients with LOS ≥ 14: 86.2% vs. 82.7% vs. 76.2% vs. 70.7%). The percentage of patients with BI < 60 was higher in the order of CRP+BMI+, CRP-BMI+, CRP+BMI-, and CRP-BMI- (percentage of patients with BI < 60: 73.9% vs. 61.4% vs. 47.7% vs. 38.1%).

In the complete case analysis, similar to the all-case analysis, there were significant differences between any two groups for BI < 60 (all *p*-values < 0.01). No significant differences were observed between the CRP+BMI- and CRP-BMI+ groups in terms of in-hospital mortality and the composite outcome (*p* = 0.22, and *p* = 0.19, respectively). There was also no significant difference between the CRP+BMI- group and the CRP+BMI+ group in terms of LOS ≥ 14 (*p* = 0.04). There was a significant difference between the CRP+BMI+ group and the other groups, excluding LOS ≥ 14, as well as between the CRP-BMI- group and the other groups (all *p* values < 0.01). In-hospital mortality and the percentage of patients with LOS ≥ 14 were higher in the order of CRP+BMI+, CRP+BMI-, CRP-BMI+, and CRP-BMI- (in-hospital mortality: 24.7% vs. 16.1% vs. 14.8% vs. 7.5%; percentage of patients with LOS ≥ 14: 89.4% vs. 87.2% vs. 82.5% vs. 76.6%). The percentage of patients with BI < 60 was higher in the order of CRP+BMI+, CRP-BMI+, CRP+BMI-, and CRP-BMI- (percentage of patients with BI < 60: 69.6% vs. 55.4% vs. 45.3% vs. 31.3%) (Supplementary Table S3).

## 4. Discussion

The present results suggest the utility of the maximum CRP level within the first three days as a more appropriate assessment of inflammatory status. The optimal cut-off level for CRP was 3.82 mg/dL for all the patients but varied depending on diagnoses. The use of this optimal cut-off as an inflammation criterion and combining it with the low BMI criteria of the GLIM phenotypic criteria enabled us to identify those patients with malnutrition who had significantly higher mortality rates, longer LOS, and worse activities of daily living at discharge. To the best of our knowledge, the prognoses of patients have not yet been examined based on the maximum CRP level several days after admission. In addition, the relationship between patient prognosis and a nutritional assessment that combines the maximum CRP level with BMI has yet to be investigated. BMI reflects nutritional status, and a low BMI is associated with a poor prognosis [20,21]. When combined with CRP, it reflects the severity of physiological stress and the inflammatory response. This aligns with the GLIM framework, which integrates phenotypic and etiologic criteria—each serving as an independent prognostic factor while also exerting a synergistic effect. Furthermore, BMI has been shown to correlate positively with CRP [22]. A low BMI combined with high CRP may strongly indicate either severe malnutrition or a high inflammatory burden, thereby identifying those patients with the poorest prognoses. Based on the results obtained herein, we propose that, when using CRP as an inflammatory etiologic criterion in the GLIM, the maximum level within 3 days after ICU admission needs to be considered. Furthermore, the results of the present study showed that it is possible to identify patients with poor prognoses by focusing on the maximum CRP level between the first 3 days and using an appropriate cut-off. Therefore, it is possible to use this cut-off in order to identify inflammation as positive at the time of a nutritional assessment without having to wait 48 h.

One of the purposes of a nutritional assessment is to identify patients who are at a high risk of malnutrition in the future. One of the assessment items used is inflammatory status. Although malnutrition is known to be caused by diseases, it may also be attributed to a lack of nutrition intake or impaired anabolism by immobilization [8]. Acute or chronic inflammation of various degrees may lead to changes in body composition and a decline in physical functions [23,24]. Furthermore, all critically ill patients may be at risk of inflammation due to the severe disease that led to their admission to the ICU. However, the results of the present study showed that CRP contributed to the identification of patients with poor prognoses and supported the assessment of inflammation using the GLIM on an individual basis, even for critically ill patients. The present results indicate that this use of CRP is particularly meaningful in conjunction with a nutritional assessment including BMI.

Markers in the GLIM are used to recognize inflammation, and the criteria applied to each condition remain unclear. IL-6 has been proposed as an excellent indicator of inflammation, including the NUTRIC score [25,26]. IL-6 has the advantage of being a sensitive inflammation marker that correlates well with the prognoses of patients; however, if the time of the assessment is delayed from disease onset or the peak of disease activity, the value will significantly decrease because of its half-life [12]. On the other hand, changes in CRP lag behind the actual inflammatory response. There are cases in which the CRP level is already high at the time of hospitalization, as in the case of sepsis a few days after onset, or when it reaches its peak within a few days, such as following surgery. CRP levels begin to rise 4–6 h after an invasion and peak at around 36 h [12], making it difficult to assess inflammation during the hyperacute phase. Nevertheless, we believe that the methodology proposed in this study—evaluating the maximum CRP between days 0 and 2—mitigates this limitation and enables a more accurate assessment of acute inflammation. The risk of overestimating inflammation beyond the acute phase is also a concern due to the slow resolution of CRP levels; however, this is not a major problem in the initial nutritional assessment. The use of the maximum CRP level over several days, as in the present study, is considered to reflect actual clinical practice. CRP has been evaluated as a reliable indicator of the prognoses of chronic diseases [27,28,29], as well as some diseases in the acute phase [30,31,32]. Since blood tests are often performed every day during an ICU stay, CRP levels may be applied to detect inflammation in a nutritional assessment, particularly in critically ill patients.

In the present study, the optimal cut-off level for CRP markedly varied depending on the diagnoses. The purpose of the present study and its interpretation were to clarify how to use CRP with a focus on the timing of the evaluation of inflammation in a nutritional assessment, not to identify the optimal cut-off level for CRP. In the present ICU cohort, we demonstrated that it was possible to identify patients with poor prognoses by combining the BMI criteria in the GLIM and an inflammation criterion using a cut-off level for CRP of 3–4 mg/dL. However, based on variations in the optimal cut-off values for each diagnosed disease, more accurate selection of patients at risk may be achieved by setting a cut-off CRP level for each diagnosed disease. The CRP levels were often high in cases of sepsis and digestive diseases that may comprise a number of infectious and inflammatory diseases, and the optimal cut-off level for CRP was slightly higher. The study’s target population comprised patients urgently admitted to the ICU; therefore, we considered infectious and inflammatory diseases to be more common in the sepsis and gastrointestinal categories, while infections were less common in the neurological and other disease categories. However, many of the diseases in these categories are associated with severe inflammation, and, thus, these patients are considered to be at risk of inflammation regardless of their CRP levels. On the other hand, in neurology and other diagnosed diseases, the CRP levels are generally not high; therefore, even with low CRP levels, there is a risk of inflammation. Although disease-specific CRP cut-offs may be too complex for general medical staff to implement in daily nutritional assessments, our findings indicated the difficulties in setting universal cut-offs. Physicians in various departments, as well as those in coronary care and stroke units, often treat patients with specific diseases or conditions. Therefore, introducing disease-specific CRP cut-off values may enhance the accuracy of identifying malnourished patients with poor prognoses. Such cut-offs may also enable more detailed assessments by nutritionists and nutrition teams. If CRP is only used for screening and not for the severity grading of malnutrition, it may be reasonable for clinical practice to use a rough and low cut-off value, such as a CRP value of 3–4 mg/dL, in critically ill patients. This CRP cut-off makes it easier to include high-inflammation diseases, such as sepsis.

In summary, the present study offered valuable insights into the potential role of CRP in nutritional assessments for critically ill patients. The maximum CRP value within the first three days of admission in the GLIM etiologic criteria enables a nutritional assessment to identify patients with poor prognoses. This method enables a nutritional assessment to be conducted earlier if CRP exceeds the cut-off value on days 0 or 1. The current study suggested a cut-off level of 3–4 mg/dL, but it also noted the challenges of establishing a universal cut-off for different diseases.

There are several limitations that need to be addressed. External validation was not performed in the present study, and the results may have been influenced by the center-dependent patient population in Japan. To overcome this problem with external validity, prospective multicenter studies are required. Our exclusion criteria (e.g., missing data for discharge BI, BMI, or age) may have introduced potential selection bias in our findings. Furthermore, missing CRP levels were linearly interpolated, which may not have reflected actual changes. Nevertheless, CRP has a long half-life and changes gradually over time; therefore, linear interpolation may be reasonable, and the bias from this imputation method was considered to be minimal. Additionally, in the present study, we were only able to assess BMI as part of the GLIM phenotypic criteria due to dataset limitations, which may introduce bias. However, our subjects (patients emergently admitted to ICU) were in the acute phase on admission; therefore, many of them did not experience weight loss one or more months before admission. In fact, several previous studies have utilized BMI alone as a prognostic indicator in nutritional assessments [33,34]. Another limitation is the discrepancy in the optimal cut-off level for CRP between the all-case analysis and complete case analysis. The percentage of patients categorized under neurology was higher in the all-case analysis, and the patients in the complete case analysis were more severe than those in the all-case analysis. The present results suggest that the optimal cut-off may depend on the diagnosis, and it may vary for all patients depending on the composition and severity of the diseases resulting in their admission to the ICU.

## 5. Conclusions

In critically ill patients, the GLIM assessment using the maximum CRP level within 3 days after ICU admission may be useful for identifying patients with malnutrition risks. The present study suggested the optimal cut-off level of 3–4 mg/dL; however, the difficulties in setting a universal cut-off for various diseases were also indicated. Further investigations are warranted to conduct external validation and to evaluate the clinical implications of disease-specific cut-off values, including their relative benefits and limitations.

## Figures and Tables

**Table 1:** Area under the curve of the maximum C-reactive protein level (day 0, days 0–1, and days 0–2) for all patients and subgroups stratified by diagnoses in all/complete case analyses.

	All-Case Analysis				Complete Case Analysis			
Diagnosed Disease	AUC Using CRP (Day 0)	AUC Using Max CRP (Days 0–1)	AUC Using Max CRP (Days 0–2)	Optimal Cut-off (mg/dL)	AUC Using CRP (Day 0)	AUC Using Max CRP (Days 0–1)	AUC Using Max CRP (Days 0–2)	Optimal Cut-Off (mg/dL)
All	0.59	0.63	0.65	3.82	0.59	0.62	0.65	8.09
Sepsis	0.58	0.59	0.62	15.65	0.59	0.60	0.66	20.60
Cardiovascular	0.64	0.67	0.67	3.81	0.64	0.67	0.65	2.11
Pulmonary	0.68	0.69	0.70	5.52	0.69	0.63	0.65	8.85
Metabolic	0.63	0.65	0.65	2.16	0.68	0.69	0.69	4.02
Neurology	0.60	0.72	0.75	0.76	0.54	0.64	0.67	2.55
Trauma	0.62	0.69	0.71	4.19	0.61	0.65	0.65	6.72
Digestive	0.64	0.63	0.65	10.29	0.62	0.62	0.63	15.46
Others	0.52	0.60	0.67	5.27	0.48	0.53	0.63	10.30

The optimal cut-off was calculated using the highest AUC value out of CRP day 0, max CRP between days 0 and 1, and max CRP between days 0 and 2. Abbreviations: AUC, area under the curve; CRP, C-reactive protein.

**Table 2:** Clinical characteristics of patients divided into groups based on the low body mass index criteria (<18.5 for <70 years and <20 for ≥70 years) and C-reactive protein criterion (>3.82 mg/dL) * in the all-case analysis.

	Overall	CRP+BMI+	CRP-BMI+	CRP+BMI-	CRP-BMI-
Variables	n = 38,981	n = 6059	n = 3989	n = 15,888	n = 13,045
Age, mean (SD), years	73.1 (14.5)	79.3 (11.9)	79.6 (12.5)	71.0 (14.6)	70.7 (14.7)
Male, n (%)	22,904 (58.8%)	3291 (54.3%)	1651 (41.4%)	10,305 (64.9%)	7657 (58.7%)
BMI, mean (SD), kg/m^2^	22.5 (4.6)	17.4 (1.8)	17.6 (1.7)	24.3 (4.2)	24.1 (3.8)
Smoker, n (%)	12,670 (38.1%)	1744 (33.4%)	942 (27.3%)	5634 (41.9%)	4349 (39.2%)
Emergent surgery, n (%)	5806 (14.9%)	1129 (18.6%)	198 (5.0%)	3618 (22.8%)	861 (6.6%)
SOFA score, mean (SD)	4.0 (3.2)	4.9 (3.4)	3.1 (2.5)	4.9 (3.5)	2.6 (2.4)
CHDF, n (%)	1317 (3.4%)	237 (3.9%)	45 (1.1%)	888 (5.6%)	147 (1.1%)
IHD, n (%)	1135 (2.9%)	173 (2.9%)	119 (3.0%)	491 (3.1%)	352 (2.7%)
Mechanical ventilation, n (%)	10,045 (25.8%)	2010 (33.2%)	619 (15.5%)	5696 (35.9%)	1720 (13.2%)
ECMO, n (%)	1313 (3.4%)	200 (3.3%)	21 (0.5%)	1011 (6.4%)	81 (0.6%)
IABP, n (%)	1553 (4.0%)	123 (2.0%)	34 (0.9%)	1120 (7.0%)	1720 (13.2%)
Catecholamine index, mean (SD)	2.1 (6.7)	3.9 (9.8)	0.9 (4.0)	3.0 (7.6)	0.5 (2.8)
CRP values, mean (SD), mg/dL					
Day 0	4.4 (7.5)	7.6 (8.2)	0.7 (0.9)	7.2 (9,1)	0.6 (0.8)
Day 1	6.0 (7.8)	10.1 (7.8)	0.9 (0.9)	9.9 (8.7)	0.8 (0.9)
Day 2	7.6 (8.3)	12.3 (7.7)	1.2 (1.1)	12.7 (8.3)	1.1 (1.1)
Max between days 0 and 1	6.2 (8.3)	10.6 (8.3)	1.0 (0.9)	10.3 (9.2)	0.8 (0.9)
Max between days 0 and 2	8.4 (9.2)	13.6 (8.4)	1.3 (1.1)	14.0 (9.1)	1.2 (1.1)
Diagnosed disease at ICU admission, n (%)					
Sepsis	4457 (11.4%)	1373 (22.7%)	139 (3.5%)	2686 (16.9%)	259 (2.0%)
Cardiovascular	9608 (24.6%)	985 (16.3%)	995 (24.9%)	4089 (25.7%)	3539 (27.1%)
Pulmonary	2476 (6.4%)	854 (14.1%)	287 (7.2%)	1005 (6.3%)	330 (2.5%)
Metabolic	784 (2.0%)	141 (2.3%)	143 (3.6%)	253 (1.6%)	247 (1.9%)
Neurology	10,721 (27.5%)	820 (13.5%)	1614 (40.5%)	2247 (14.1%)	6040 (46.3%)
Trauma	2923 (7.5%)	487 (8.0%)	296 (7.4%)	1369 (8.6%)	771 (5.9%)
Digestive	1896 (4.9%)	450 (7.4%)	146 (3.7%)	866 (5.5%)	434 (3.3%)
Others	6116 (15.7%)	949 (15.7%)	369 (9.3%)	3373 (21.2%)	1425 (10.9%)

* If the low BMI criteria were met, they were represented as BMI+, and, if the CRP criterion was met, it was represented as CRP+. Abbreviations: SD, standard deviation; BMI, body mass index; SOFA, Sequential Organ Failure Assessment; CHDF, continuous hemodialysis and filtration; IHD, intermittent infusion hemodialysis; ECMO, extracorporeal membrane oxygenation; IABP, intra-aortic balloon pumping; CRP, C-reactive protein; max, maximum; ICU, intensive care unit.

**Table 3:** Comparison of outcomes in four groups based on the low body mass index criteria (<18.5 for <70 years and <20 for ≥70 years) and C-reactive protein criterion (>3.82 mg/dL) * in the all-case analysis.

	CRP+BMI+	CRP-BMI+	CRP+BMI-	CRP-BMI-
Outcome	n = 6059	n = 3989	n = 15,888	n = 13,045
Primary outcome				
In-hospital mortality, n (%)	1375 (22.7%)	430 (10.8%)	2288 (14.4%)	622 (4.8%)
Barthel index at discharge < 60, n (%)	4475 (73.9%)	2448 (61.4%)	7585 (47.7%)	4972 (38.1%)
Length of hospital stay ≥ 14, n (%)	4708 (86.2%)	2904 (76.2%)	12,235 (82.7%)	9012 (70.7%)
Composite outcome, n (%)	5753 (94.9%)	3469 (87.0%)	14,019 (88.2%)	10,018 (76.8%)
Secondary outcome				
14-day mortality, n (%)	599 (9.9%)	180 (4.5%)	1097 (6.9%)	300 (2.3%)
28-day mortality, n (%)	952 (15.7%)	298 (7.5%)	1685 (10.6%)	445 (3.4%)
Barthel index at discharge, median (IQR)	22.5 (0, 80)	50 (5, 95)	85 (20, 100)	90 (40, 100)
Length of hospital stay, median (IQR), days	25 (15, 42)	21 (13, 34)	23 (14, 39)	19 (12, 31)

* If the low BMI criteria were met, they were represented as BMI+, and, if the CRP criterion was met, it was represented as CRP+. The results of Kruskal–Wallis tests were significant for all outcomes (all *p*-values < 0.01). Conover tests revealed that there were significant differences between any two groups for in-hospital mortality, BI < 60, and LOS ≥ 14 (all *p*-values < 0.01). There was no significant difference between the CRP+BMI- group and CRP-BMI+ group in terms of the composite outcome (*p* = 0.04). Abbreviations: IQR, interquartile range; CRP, C-reactive protein; BMI, body mass index.

## Data Availability

Publicly available datasets were analyzed in this study. This data can be found here: The administrative claims database of inpatients and laboratory test results in Japan, supplied by Medical Data Vision Co., Ltd. (Tokyo, Japan), was used in this retrospective cohort study. The datasets generated and analyzed during the present study are available from the corresponding author upon reasonable request.

## Supplementary Materials

The following supporting information can be downloaded at https://www.mdpi.com/article/10.3390/nu17040705/s1. Supplementary Table S1: The sensitivity and specificity when the C-reactive protein cut-off was changed for each diagnosed disease. Supplementary Table S2: Clinical characteristics of patients divided into groups based on the low body mass index criteria (<18.5 for <70 years and <20 for ≥70 years) and C-reactive protein criterion (>8.09 mg/dL) * in the complete case analysis. Supplementary Table S3: Comparison of outcomes in four groups based on the low body mass index criteria (<18.5 for <70 years and <20 for ≥70 years) and C-reactive protein criterion (>8.09 mg/dL) * in the complete case analysis.

## Abbreviations

The following abbreviations are used in this manuscript:

GLIM	Global Leadership Initiative on Malnutrition
CRP	C-reactive protein
ICU	Intensive care unit
AUC	Area under the curve
BMI	Body mass index
NUTRIC	Nutrition Risk in Critically Ill
IL-6	Interleukin-6
BI	Barthel index
CHDF	Continuous hemodialysis and filtration
ECMO	Extracorporeal membrane oxygenation
IABP	Intra-aortic balloon pumping
SOFA	Sequential Organ Failure Assessment
ROC	Receiver operating characteristic
