# Supplementary material for: Use of C-Reactive Protein in Global Leadership Initiative on Malnutrition (GLIM) Etiologic Criteria for Critically Ill Patients: A Retrospective Claims Database Study
Source: Nutrients. 2025 Feb 16;17(4):705. doi: 10.3390/nu17040705 (PMC11858327; doi:10.3390/nu17040705)
Supplement: Supplementary file 1 [file nutrients-17-00705-s001.zip › nutrients-3469361-supplementary.pdf]

Supplementary

Table S1. The sensitivity and specificity when the CRP cut-off was changed for each diagnosed disease.

	Overall (N=38,981)		Sepsis (N=4,457)		Cardiovascular (N=9,608)		Pulmonary (N=24,76)		Metabolic (N=784)		Neurology (N=10,721)		Trauma (N=2,923)		Digestive (N=1,896)		Others (N=6,116)	
CRP cut-off (mg/dL)	Se	Sp	Se	Sp	Se	Sp	Se	Sp	Se	Sp	Se	Sp	Se	Sp	Se	Sp	Se	Sp
2.5	0.67	0.54	0.95	0.1	0.67	0.55	0.84	0.45	0.65	0.6	0.4	0.92	0.77	0.55	0.78	0.39	0.82	0.4
3	0.64	0.57	0.94	0.11	0.63	0.6	0.81	0.49	0.61	0.63	0.36	0.94	0.74	0.58	0.75	0.4	0.79	0.44
3.5	0.61	0.6	0.92	0.13	0.6	0.65	0.79	0.5	0.57	0.66	0.33	0.94	0.71	0.61	0.73	0.44	0.77	0.48
3.82	0.59	0.62	0.92	0.14	0.58	0.67	0.77	0.52	0.55	0.67	0.31	0.94	0.69	0.64	0.73	0.46	0.75	0.5
4	0.58	0.63	0.91	0.15	0.56	0.68	0.76	0.52	0.53	0.68	0.3	0.95	0.68	0.65	0.72	0.46	0.74	0.51
4.5	0.56	0.65	0.9	0.17	0.53	0.71	0.74	0.55	0.5	0.69	0.27	0.95	0.65	0.68	0.7	0.48	0.72	0.53

Abbreviations: CRP, C-reactive protein; Se, Sensitivity; Sp, Specificity.

Table S2. Clinical characteristics of patients divided into groups based on the low body mass index criteria (<18.5 for <70 years and <20 for ≥70 years) and C-reactive protein criterion (>8.09 mg/dL) * in the complete case analysis.

Variables	Overall n=12,536	CRP+BMI+ n=1808	CRP-BMI+ n=1227	CRP+BMI- n=5588	CRP-BMI- n=3913
Age, mean (SD), years	72.2 (14.5)	78.1 (12.2)	77.9 (12.5)	70.6 (14.4)	69.8 (14.8)
Male, n (%)	7717 (62%)	974 (53.9%)	559 (45.6%)	3703 (66.3%)	2481 (63.4%)
BMI, mean (SD), kg/m2	22.7 (4.9)	17.6 (1.8)	17.6 (1.7)	24.4 (4.3)	24.3 (4.5)
Smoker, n (%)	4053 (40%)	499 (33.5%)	304 (31.5%)	1920 (41.7%)	1330 (43%)
Emergent surgery, n (%)	2772 (22.1%)	564 (31.2%)	122 (9.9%)	1643 (29.4%)	443 (11.3%)
SOFA score, mean (SD)	5.2 (3.6)	6.2 (3.7)	4.4 (3.2)	5.9 (3.8)	4.0 (3.2)
CHDF, n (%)	956 (7.6%)	169 (9.3%)	44 (3.6%)	575 (10.3%)	168 (4.3%)
IHD, n (%)	394 (3.1%)	54 (3.0%)	44 (3.6%)	169 (3%)	127 (3.2%)
Mechanical ventilation, n (%)	4795 (38.2%)	819 (45.3%)	376 (30.6%)	2541 (45.5%)	1059 (27.1%)
ECMO, n (%)	856 (6.8%)	91 (5.0%)	47 (3.8%)	510 (9.2%)	208 (5.3%)
IABP, n (%)	929 (7.4%)	53 (2.9%)	35 (2.9%)	515 (9.2%)	326 (8.3%)
Catecholamine index, mean (SD)	3.8 (8.7)	7.0 (12.5)	2.4 (7.3)	4.6 (9.0)	1.7 (5.2)
CRP values, mean (SD), mg/dL					
Day 0	5.5 (8.7)	8.9 (9.3)	1.3 (1.8)	8.5 (10.5)	1.1 (1.6)
Day 1	8.3 (9.0)	12.7 (8.6)	2.2 (2.1)	12.6 (9.7)	1.9 (1.8)
Day 2	11.6 (9.5)	17.1 (7.9)	3.4 (2.4)	17.3 (8.5)	3.5 (2.4)
Max between day 0–1	8.7 (9.6)	13.4 (9.2)	2.3 (2.1)	13.3 (10.4)	2.0 (1.9)
Max between day 0–2	12.8 (10.3)	18.8 (8.2)	3.7 (2.5)	19.2 (9.1)	3.7 (2.4)
Diagnosed disease at ICU admission, n (%)					
Sepsis	2053 (16.4%)	535 (29.6%)	73 (5.9%)	1298 (23.2%)	147 (3.8%)
Cardiovascular	4135 (33.0%)	308 (17.0%)	458 (37.3%)	1484 (26.6%)	1885 (48.2%)
Pulmonary	537 (4.3%)	144 (8.0%)	67 (5.5%)	252 (4.5%)	74 (1.9%)
Metabolic	341 (2.7%)	40 (2.2%)	76 (6.2%)	104 (1.9%)	121 (3.1%)
Neurology	1021 (8.1%)	99 (5.5%)	163 (13.3%)	285 (5.1%)	474 (12.1%)
Trauma	868 (6.9%)	117 (6.5%)	94 (7.7%)	423 (7.6%)	234 (6.0%)
Digestive	1010 (8.1%)	234 (12.9%)	102 (8.3%)	427 (7.6%)	247 (6.3%)

Others	2571 (20.5%)	331 (18.3%)	194 (15.8%)	1315 (23.5%)	731 (18.7%)

* If the low BMI criterion was met, it was represented as BMI+, and if the CRP criterion was met, it was represented as CRP+. Abbreviations: SD, Standard deviation; BMI, Body mass index; SOFA, Sequential organ failure assessment; CHDF, Continuous hemodialysis and filtration; IHD, Intermittent infusion hemodialysis; ECMO, Extracorporeal membrane oxygenation; IABP, Intra-aortic balloon pumping; CRP, C-reactive protein; max, maximum; ICU, Intensive care unit.

Table S3. Comparison of outcomes in four groups based on the low body mass index criteria (<18.5 for <70 years and <20 for ≥70 years) and C-reactive protein criterion (>8.09 mg/dL) * in the complete case analysis.

	CRP+BMI+	CRP-BMI+	CRP+BMI-	CRP-BMI-
Outcome	n=1808	n=1227	n=5588	n=3913
Primary outcome				
In-hospital mortality, n (%)	446 (24.7%)	181 (14.8%)	900 (16.1%)	292 (7.5%)
Barthel index at discharge < 60, n (%)	1259 (69.6%)	680 (55.4%)	2535 (45.3%)	1226 (31.3%)
Length of hospital stay ≧ 14, n (%)	1444 (89.4%)	945 (82.5%)	4527 (87.2%)	2900 (76.6%)
Composite outcome, n (%)	1737 (96.1%)	1105 (90.1%)	5105 (91.4%)	3179 (81.2%)
Secondary outcome				
14-day mortality, n (%)	192 (10.6%)	81 (6.6%)	398 (7.1%)	125 (3.2%)
28-day mortality, n (%)	297 (16.4%)	133 (10.8%)	634 (11.3%)	210 (5.4%)
Barthel index at discharge, median (IQR)	40 (0,95)	60 (10,100)	95 (30,100)	100 (55,100)
Length of hospital stay, median (IQR), days	27 (16,47)	23 (14,37)	25 (16,43)	20 (13,34)

* If the low BMI criterion was met, it was represented as BMI+, and if the CRP criterion was met, it was represented as CRP+. The results of Kruskal-Walis tests were significant for all outcomes (all p-values <0.01). Conover tests revealed that there were significant differences between any two groups for in-hospital mortality, BI <60, and LOS ≥14 (all p-values <0.01). There was no significant difference between the CRP+BMI- group and CRP-BMI+ group in terms of the composite outcome (p=0.04).

Abbreviations: IQR, Interquartile range; CRP, C-reactive protein; BMI, Body mass index.
